# Electrical impedance tomography for titration of positive end-expiratory pressure in acute respiratory distress syndrome patients with chronic obstructive pulmonary disease

**DOI:** 10.1186/s13054-022-04201-y

**Published:** 2022-11-04

**Authors:** Xuesong Liu, Xiao Liu, Jue Meng, Dongdong Liu, Yongbo Huang, Ling Sang, Yonghao Xu, Zhiheng Xu, Weiqun He, Sibei Chen, Rong Zhang, Xiaoqing Liu, Yimin Li

**Affiliations:** 1 State Key Lab of Respiratory Diseases, Guangzhou Institute of Respiratory Health, Department of Critical Care Medicine, The First Affiliated Hospital of Guangzhou Medical University, 151 Yanjiang Street West, Guangzhou, 510120 Guangdong China; 2 Department of Critical Care Medicine, Huadu District People’s Hospital, Guangzhou, 510800 China; 3 Department of Respiratory Medicine, Nanhai District People’s Hospital and Sixth Affiliated Hospital of South China University of Technology, Foshan, 528200 Guangdong China

**Keywords:** Acute respiratory distress syndrome, Chronic obstructive pulmonary disease, Positive end-expiratory pressure, Electrical impedance tomography, Oxygen delivery, Ventilation distribution

## Abstract

**Background:**

Chronic obstructive pulmonary disease (COPD) is one of most common comorbidities in acute respiratory distress syndrome (ARDS). There are few specific studies on the appropriate ventilation strategy for patients with ARDS comorbid with COPD, especially regarding on positive end-expiratory pressure (PEEP) titration.

**Methods:**

To compare the respiratory mechanics in mechanical ventilated ARDS patients with or without COPD and to determine whether titration of PEEP based on electrical impedance tomography (EIT) is superior to the ARDSnet protocol. This is a single center, perspective, repeated measure study. ARDS patients requiring mechanical ventilation who were admitted to the intensive care unit between August 2017 and December 2020 were included. ARDS patients were divided according to whether they had COPD into a COPD group and a non-COPD group. Respiratory mechanics, gas exchange, and hemodynamics during ventilation were compared between the groups according to whether the PEEP level was titrated by EIT or the ARDSnet protocol.

**Results:**

A total of twenty-seven ARDS patients including 14 comorbid with and 13 without COPD who met the study eligibility criteria were recruited. The PEEP levels titrated by EIT and the ARDSnet protocol were lower in the COPD group than in the non-COPD group (6.93 ± 1.69 cm H_2_O vs. 12.15 ± 2.40 cm H_2_O, *P* < 0.001 and 10.43 ± 1.20 cm H_2_O vs. 14.0 ± 3.0 cm H_2_O, *P* < 0.001, respectively). In the COPD group, the PEEP level titrated by EIT was lower than that titrated by the ARDSnet protocol (6.93 ± 1.69 cm H_2_O vs. 10.43 ± 1.20 cm H_2_O, *P* < 0.001), as was the global inhomogeneity (GI) index (0.397 ± 0.040 vs. 0.446 ± 0.052, *P* = 0.001), plateau airway pressure (16.50 ± 4.35 cm H_2_O vs. 20.93 ± 5.37 cm H_2_O, *P* = 0.001), dead space ventilation ratio (48.29 ± 6.78% vs. 55.14 ± 8.85%, *P* < 0.001), ventilation ratio (1.63 ± 0.33 vs. 1.87 ± 0.33, *P* < 0.001), and mechanical power (13.92 ± 2.18 J/min vs. 15.87 ± 2.53 J/min, *P* < 0.001). The cardiac index was higher when PEEP was treated by EIT than when it was titrated by the ARDSnet protocol (3.41 ± 0.50 L/min/m^2^ vs. 3.02 ± 0.43 L/min/m^2^, *P* < 0.001), as was oxygen delivery (466.40 ± 71.08 mL/min/m^2^ vs. 411.10 ± 69.71 mL/min/m^2^, *P* = 0.001).

**Conclusion:**

Titrated PEEP levels were lower in patients with ARDS with COPD than in ARDS patients without COPD. In ARDS patient comorbid with COPD, application of PEEP titrated by EIT was lower than those titrated by the ARDSnet protocol, which contributed to improvements in the ventilation ratio, mechanical energy, cardiac index, and oxygen delivery with less of an adverse impact on hemodynamics.

**Supplementary Information:**

The online version contains supplementary material available at 10.1186/s13054-022-04201-y.

## Introduction

The response of patients with acute respiratory distress syndrome (ARDS) to positive end-expiratory pressure (PEEP) application during mechanical ventilation is widely varied due to the lung pathological heterogeneity of ARDS, and individualized ventilation strategies tailed to the physiological characteristic of lung are expected to improve the outcome of patients with ARDS [1, 2]. Chronic obstructive pulmonary disease (COPD) is one of the most important comorbidities in patients with ARDS, accounting for up to 21% of ARDS population as shown in a observational study [3]. Small airway lesion is a well-recognized feature of COPD which is characterized by poorly reversible airflow limitation, pulmonary dynamic hyperinflation, gas trapping, and intrinsic PEEP (PEEPi) [4]. Heterogeneity in lung pathological traits and ventilation distribution is more complex in ARDS patients with COPD [5]. Furthermore, application of PEEP during mechanical ventilation raised the risk of compromised cardiac function, increased lung volume and excessive alveolar expansion which is one of major contributors to ventilator-related lung injury (VILI) [6, 7]. Therefore, ARDS patients with COPD are at greater risk for adverse effects when applying PEEP during ventilation, and the best ventilation strategy, particularly regarding optimal PEEP selection, in these patients remains inconclusive.

There are many methods available for the clinical selection of PEEP during lung protective ventilation strategies, but the appropriate method for titrating PEEP in ARDS patients with COPD remains a challenging question [8]. Electrical impedance tomography (EIT) is a recent developed method to evaluate ventilation distribution in the lung [9] which can monitor the intrathoracic ventilation distribution and local and global respiratory mechanic reflected by impedance changes in response to ventilation [10, 11]. Besides, EIT has the advantages of being non-invasive, radiation-free and allowing real-time monitoring at the bedside. Titration of PEEP guided by EIT should achieve the best compromise between lung collapse and overdistension. Recent studies have proved the safety and feasibility of PEEP titration guided by EIT at the bedside in ventilated patients with ARDS. Compared with other methods, titration of the PEEP level by EIT leaded to more uniform ventilation distribution, better respiratory mechanics and higher oxygenation in mechanically ventilated patients as well as improved clinical outcomes [5, 10, 12–14]. However, there is limited research focusing on titration of PEEP guided by EIT in ARDS patients with COPD. It is unknown whether the PEEP level required for ventilated ARDS patients with COPD differs from that required in ARDS patients without COPD. Therefore, we hypothesized that (1) the optimal PEEP level required for ventilation in patients with ARDS may depend on whether they have COPD as a comorbidity and (2) the titrated PEEP level guided by EIT may be superior to that titrated using the traditional ARDSnet PEEP table.

## Materials and methods

### Study design and patients

This is a prospective, single-center, repeated-measures cohort study which included patients with ARDS who were admitted to the Department of Critical Care Medicine of the First Affiliated Hospital of Guangzhou Medical University in China and required mechanical ventilation during August 1, 2017, and December 31, 2020. The patients were divided into COPD group and non-COPD group according to whether they had COPD or not. The study was approved by the Ethics Committee of the First Affiliated Hospital of Guangzhou Medical University.

The inclusion criteria in both study groups were as follows: diagnostic criteria for ARDS met according to the 2012 Berlin definition [15]; invasive mechanical ventilation within  3 days and with expected duration  longer than 3 days; age older than 18 years and younger than 85 years; and signed informed consent able to be obtained. Patients in the COPD group were required to have COPD diagnosed as a comorbidity by two independent pulmonologists in accordance with medical history, pulmonary function, flow characteristics of mechanical ventilation, chest radiograph, and a pre-existing pulmonologist-confirmed diagnosis of COPD [5]. Description of clinical characteristics of COPD patients is shown in Additional file 1: Table S1.

The following exclusion criteria were applied: hemodynamically unstable or active bleeding; severe neurological disease; within 2 weeks post-lung surgery; pregnancy or lactation; end-stage malignant disease; history of organ transplantation; contraindication to use of EIT; and critical illness with an expected survival time of less than 48 h.

### Titration of PEEP

All patients were fully sedated and received analgesics and muscle relaxants. The patients were ventilated at supine position with mechanical ventilation settings as follows: volume control mode; tidal volume, 6 mL/kg predicted body weight; PEEP, 5 cm H_2_O; plateau pressure, < 30 cm H_2_O. An arteriovenous catheter was placed, and EIT was connected in all cases.

### ARDSnet protocol

While maintaining SpO_2_ at 88%–95%, the PEEP level was selected according to the FiO_2_/PEEP lower table method. The patients were ventilated 30 min with PEEP selected by ARDSnet method, and then, parameters regarding respiratory mechanics, hemodynamics, mechanical distribution, gas exchange and oxygen delivery were measured or calculated.

### EIT method

To find the optimal PEEP under the guidance of EIT, keeping other ventilation parameters unchanged, we firstly set the PEEP at 5 cm H_2_O and then increased the PEEP level from 5 to 8 cm H_2_O, 10 cm H_2_O, 12 cm H_2_O, 14 cm H_2_O, 16 cm H_2_O, 18 cm H_2_O, 20 cm H_2_O every 30 min. Considering higher PEEP level causes marked decrease in cardiac output in patients with cardiac disease [16] and COPD is a risk factor for right ventricular dysfunction [17], we did not further increase the PEEP level when it reached 16 cm H_2_O to avoid the potential risk of hemodynamic instability in the COPD group. At the end of every 30-min ventilation, parameters of respiratory mechanics, hemodynamics, mechanical distribution, gas exchange and oxygen delivery were measured and calculated. We then obtained the global inhomogeneity (GI) index for the whole lung and the PEEP which is corresponding to the minimum GI index value (the lowest point of the curve) recorded by EIT was defined as best PEEP titrated by EIT based on the GI index.

The indications for termination of the experiment were as follows: blood pressure < 80/60 mmHg, pulse rate > 120 beats/min, and a need for a substantial increase in the vasopressor dose to maintain circulatory stability (e.g., a cardiac index < 1.5 L/min/m^2^); a decrease in peripheral blood oxygen saturation (SpO_2_) to < 80%; and pneumothorax, bleeding, or other clinical condition that meant the test could not be continued.

### EIT measurements

EIT measurements were performed using the PulmoVista 500 tomograph (Dräger PulmoVista 500, Lübeck, Germany). Electrical conductivity of the chest is used to generate cross-sectional images of the lung inferred from surface electrical measurements realized by a 16-electrode belt. Four horizontal parallel regions of interest (ROIs) within the chest contour were selected: ROI 1 (ventral), ROI 2 (central ventral), ROI 3 (central dorsal), and ROI 4 (dorsal). To evaluate ventilation distribution, the number calculated per ROI is the sum of impedance changes in this ROI in relation to the sum of impedance changes of the whole EIT image [18]. EIT data were continuously recorded and analyzed offline using the Dräger EIT analysis tool, version 6.1 (Dräger Medical) [19]. Centre of ventilation (CoV) was calculated as following: COV (%) = (Δ*Z* in the dorsal half of lung) × 100/(Δ*Z* in the whole lung), where ∆Z = change in impedance. This reflects the distribution of tidal ventilation along the ventral–dorsal axis, and when the bulk of the ventilation is at midpoint (COV = 50%), this represents homogeneously distributed ventilation [20].

### Statistical analysis

The primary aim of this study is to determine the difference in PEEP between ARDS patients with COPD and without COPD. However, no data are available concerning the differences in PEEP between the two groups. Therefore, the sample size calculation was based on the management experience of mechanical ventilation and patients’ characteristics of ventilation settings in our center. We usually set up the PEEP 40% lower in ARDS patients with COPD than in ARDS patients without COPD. According to previous reported ventilator settings of ARDS patients treated with invasive ventilation in our center [21], we suppose that the PEEP is 10 cm H_2_O for ARDS patients without COPD and 6 cm H_2_O for ARDS patients with COPD, with an alpha = 0.05, beta = 0.10 and standard deviation of 3, 12 patients in each group were required. As a matter of fact, for the comparison of the PEEP levels titrated by EIT or the ARDSnet protocol between ARDS group and ARDS with COPD group, the achieved sample size resulted in a power (1-β) above 90%. The sample size calculation and power analysis were conducted in an online tool (http://clincalc.com/Stats).

Continuous data that were confirmed to be normally distributed are shown as the mean ± standard deviation and compared between the two study groups using the paired *t*-test. The variance analysis was performed using random block design data, not consistent. The rank-sum test was used for data that were normally distributed or ANOVA homogeneity. All statistical analyses were performed using SPSS version 22.0 (IBM Corp., Armonk, NY, USA) and GraphPad Prism 8 (GraphPad Software Inc., San Diego, CA, USA). A *P*-value < 0.05 was considered statistically significant.

## Results

### Patient demographic and clinical characteristics

**Fig. 1:**
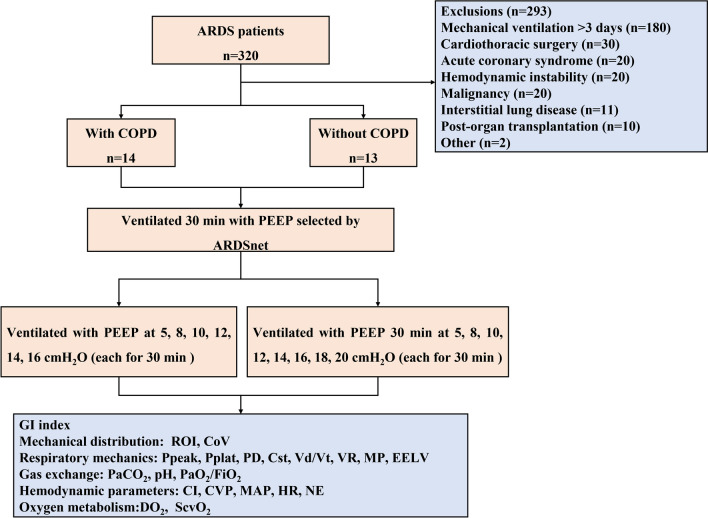
Flow chart of the study

Of 1500 patients who were mechanically ventilated in the Department of Intensive Care Medicine at the First Affiliated Hospital of Guangzhou Medical University during the study period, 320 met the diagnostic criteria for ARDS and 27 patients met the study eligibility criteria (Fig. 1). The etiology of ARDS was severe pneumonia in all cases. The mean age of patient was 70 ± 14 years, and most patients were male (*n* = 20, 74.1%). 3, 13 and 11 patients were diagnosed as severe, moderate, and mild ARDS, respectively. There were 14 ARDS patients in the COPD group and 13 in the non-COPD group. PEEPi was significantly higher in the COPD group than in the non-COPD group (5.8 ± 2.1 cm H_2_O vs. 0.31 ± 0.43 cm H_2_O, *P* < 0.001), as was the end-expiratory lung volume (EELV; 1135 ± 217.3 mL vs. 724.6 ± 130.8 mL, *P* < 0.001) (Table 1).

**Table 1:** Patient demographic and clinical characteristics

	Total (*n* = 27)	COPD group (*n* = 14)	Non-COPD group (*n* = 13)	*P*-value
Male sex (%)	20/74.1%	13/93%	7/54%	0.023
Age (years)	70 ± 14	70 ± 8	50 ± 13	< 0.001
Body mass index^a^	21.59 ± 4.56	20.4 ± 4.6	23.61 ± 4.0	0.066
PBW (kg)	60.15 ± 7.61	62.3 ± 6.7	59.3 ± 8.5	0.3193
APACHE II	21.04 ± 6.05	20.0 ± 8.0	22.2 ± 2.9	0.365
SOFA	9.27 ± 3.54	8.8 ± 3.6	9.8 ± 3.5	0.447
Murray score	2.54 ± 0.62	2.39 ± 0.59	2.69 ± 0.62	0.212
HR (bpm)	100 ± 16.69	97 ± 15	103 ± 18	0.377
MAP (mmHg)	86.85 ± 11.71	89 ± 14	84 ± 9	0.229
ScvO_2_ (%)	73.56 ± 7.953	73 ± 8	74 ± 8	0.578
P/F (mmHg)	175.5 ± 61.3	195.7 ± 47.0	153.6 ± 69.0	0.073
PEEPi	3.16 ± 3.2	5.8 ± 2.1	0.31 ± 0.43	< 0.001
EELV (mL)	937.2 ± 274	1135 ± 217.3	724.6 ± 130.8	< 0.001
ARDS				
Mild	11	7	4	
Moderate	13	6	7	
Severe	3	1	2	
Other comorbidities				
HP	7	4	3	
Type 2 DM	5	3	2	
CKD	2	1	1	
RVD	12	8	4	0.057
28-day all-cause mortality (%)	11.1	14.3	7.7	0.999
90-day all-cause mortality (%)	22.2	21.4	23.1	0.999

The data are presented as the number, number (percentage), or mean ± standard deviation

*APACHE II* Acute Physical and Chronic Health Evaluation II, *ARDS* acute respiratory distress syndrome, *BMI* body mass index, *CKD* chronic kidney disease, *DM* diabetes, *EELV* end-expiratory lung volume, *HP* hypertension, *HR* heart rate, *MAP* mean arterial pressure, *PBW* predicted body weight, *PEEP* positive end-expiratory pressure, *PEEPi* intrinsic PEEP, *P/F* PaO_2_/FiO_2_ ratio, *RVD* right ventricular dysfunction, *ScvO*_*2*_ central venous oxygen saturation, *SOFA* Sequential Organ Failure Assessment

^a^Calculated as kg/m^2^

### Comparison of PEEP level titrated by EIT method and ARDSnet protocol

**Fig. 2:**
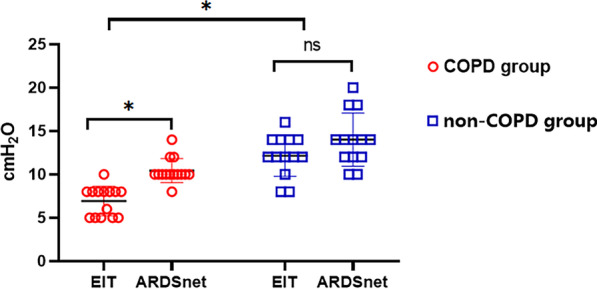
Comparison of PEEP level titrated by EIT method and ARDSnet protocol in ARDS patient with or without had COPD

The PEEP levels titrated by both EIT method based on the GI index and the ARDSnet protocol were lower in the COPD group than in the non-COPD group (EIT method: 6.93 ± 1.69 cm H_2_O vs. 12.15 ± 2.40 cm H_2_O, *P* < 0.001 and ARDSnet protocol:10.43 ± 1.20 cm H_2_O vs. 14.0 ± 3.0 cm H_2_O, *P* < 0.001, respectively). In the COPD group, the titrated PEEP level guided by EIT was lower than that titrated by the ARDSnet protocol (6.93 ± 1.69 cm H_2_O vs. 10.43 ± 1.20 cm H_2_O, *P* < 0.001). In the non-COPD group, there was no significant difference in the PEEP level titrated by EIT and that titrated by the ARDSnet protocol (12.15 ± 2.40 cm H_2_O vs. 14.0 ± 3.0 cm H_2_O, *P* = 0.098) (Fig. 2). In the COPD group, the GI index was lower when the PEEP level was titrated by EIT than when it was titrated by the ARDSnet protocol (0.397 ± 0.04 vs. 0.446 ± 0.052, *P* = 0.001); however, in the non-COPD group, there was no significant difference in the GI index according to whether the PEEP level was titrated by EIT or the ARDSnet protocol (0.45 ± 0.038 vs. 0.477 ± 0.021, *P* = 0.063) (Table 2).

**Table 2:** Comparison of the GI index between the COPD group and the non-COPD group

Group	EIT GI index	ARDSnet protocol GI index	*T*	*P*-value
COPD	0.397 ± 0.04	0.446 ± 0.052	3.571	0.001
Non-COPD	0.45 ± 0.038	0.477 ± 0.021	1.946	0.063

The data are presented as the mean ± standard deviation

*ARDS* acute respiratory distress syndrome, *COPD* chronic obstructive pulmonary disease, *EIT* electrical impedance tomography, *GI* global inhomogeneity

### Physiological comparison in the ARDS patients with COPD ventilated with PEEP titrated by ETI method and ARDSnet protocol

#### Respiratory mechanics

Peak airway pressure was significantly lower when PEEP was titrated by EIT than by the ARDSnet protocol (29.5 ± 4.1 cm H_2_O vs. 33.64 ± 5.19 cm H_2_O, *P* < 0.001), as was the airway plateau pressure (16.5 ± 4.35 cm H_2_O vs. 20.93 ± 5.37 cm H_2_O, *P* = 0.001), dead space ventilation ratio (48.29 ± 6.78% vs. 55.14 ± 8.85%, *P* < 0.001), and ventilation ratio (VR; 1.63 ± 0.33 vs. 1.87 ± 0.33, *P* < 0.001). Mechanical power was also lower when using EIT than when using the ARDSnet protocol (13.92 ± 2.18 J/min vs. 15.87 ± 2.53 J/min, *P* < 0.001). There was no significant difference in other respiratory mechanics parameter including driving pressure, when patients’ ventilation with PEEP selected by EIT method and ARDSnet protocol (*P* > 0.05) (Table 3). There is no significant difference in pulmonary shunt fraction when patients ventilated with PEEP titrated by EIT or by the ARDSnet protocol (17.8 ± 7.16% vs. 16.5 ± 4.46%, *P* = 0.352).

**Table 3:** Effects of PEEP level on respiratory and circulatory function in patients with acute respiratory distress syndrome and chronic obstructive pulmonary disease according to the titration method used

Respiratory mechanics and hemodynamic parameters	EIT	ARDSnet protocol	*T*	*P*-value
P_peak_ (cm H_2_O)	29.5 ± 4.10	33.64 ± 5.19	6.340	<0.001
P_plat_ (cm H_2_O)	16.5 ± 4.35	20.93 ± 5.37	4.030	0.001
PD (cm H_2_O)	10.0 ± 3.51	10.57 ± 4.6	0.597	0.562
Cst (mL/cm H_2_O)	42.61 ± 13.77	38.39 ± 13.86	2.186	0.049
Vd/Vt (%)	48.29 ± 6.78	55.14 ± 8.85	4.686	<0.001
VR	1.63 ± 0.33	1.87 ± 0.33	7.373	<0.001
MP (J/min)	13.92 ± 2.18	15.87 ± 2.53	5.15	<0.001
EELV (mL)	1326 ± 201.5	1440 ± 182.6	2.791	0.019
Qs/Qt (%)	17.8 ± 7.16	16.5 ± 4.46	0.966	0.352
pH	7.39 ± 0.06	7.39 ± 0.07	0.219	0.836
PaCO_2_ (mmHg)	54.07 ± 6.78	63.21 ± 8.26	7.054	<0.001
P/F (mmHg)	220.6 ± 67.73	237.2 ± 73.01	1.749	0.105
CI (L/min/m^2^)	3.41 ± 0.50	3.02 ± 0.43	4.774	<0.001
CVP (cm H_2_O)	12.12 ± 4.69	13.43 ± 3.88	1.745	0.105
MAP (mmHg)	78.5 ± 7.3	82.1 ± 12.4	1.220	0.244
HR (bpm)	81.7 ± 15.8	80.8 ± 14.7	0.618	0.547
NE (µg/kg/min)	0.105 ± 0.077	0.119 ± 0.076	2.590	0.022
DO_2_ (mL/min/m^2^)	466.4 ± 71.08	411.1 ± 69.71	3.997	0.001
ScvO_2_ (%)	71.08 ± 9.29	69.7 ± 8.09	1.029	0.322

The data are presented as the mean ± standard deviation

*ARDS* acute respiratory distress syndrome, *CI* cardiac index, *COPD* chronic obstructive pulmonary disease, *Cst* static compliance, *CVP* central venous pressure, *DO*_*2*_ oxygen delivery, *EELV* end-expiratory lung volume, *EIT* electrical impedance tomography, *HR* heart rate, *MAP* mean arterial pressure, *MP* mechanical power, *NE* norepinephrine, *PaCO*_*2*_ partial pressure of carbon dioxide in arterial blood, *PD* driving pressure, *PEEP* positive end-expiratory pressure, *P/F, PaO*_*2*_*/FiO*_*2*_* ratio* p_peak_, peak airway pressure, *P*_*plat*_ plateau airway pressure, *Qs/Qt* pulmonary shunt fraction, *Vd/Vt* dead space ventilation ratio, *ScvO*_*2*_ central venous oxygen saturation, *VR* ventilation ratio

#### Gas exchange

PaCO_2_ was significantly lower when PEEP was selected by EIT method than by the ARDSnet protocol (54.07 ± 6.78 mmHg vs. 63.21 ± 8.26 mmHg, *P* < 0.001). However, there were no significant differences in pH and PaO_2_/FiO_2_ between two titration methods used (*P* > 0.05) (Table 3).

#### Hemodynamic parameters

The cardiac index was higher when PEEP was titrated by EIT than by the ARDSnet protocol (3.41 ± 0.50 L/min/m^2^ vs. 3.02 ± 0.43 L/min/m^2^, *P* < 0.001). The norepinephrine dose administered was lower when patient was ventilation with PEEP selected by EIT method than with the ARDSnet protocol (0.105 ± 0.077 µg/kg/min vs. 0.119 ± 0.076 µg/kg/min, *P* = 0.022). Other hemodynamic parameters including central venous pressure, mean arterial pressure, and heart rate showed no difference between two titration method used (*P* > 0.05) (Table 3).

#### Ventilation distribution

**Fig. 3:**
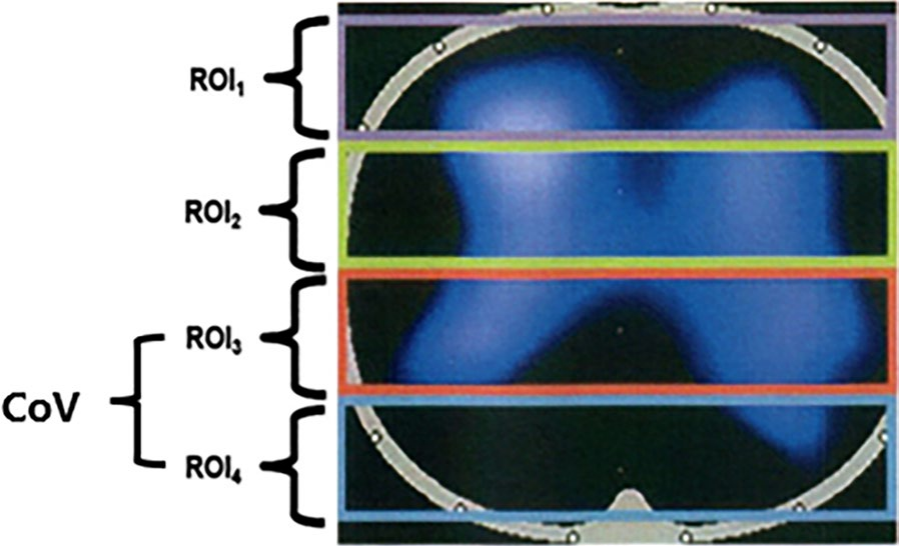
Schematic diagram showing spatial ventilation distribution

Ventilation distribution was lower in ROI (region of interest) 3% when PEEP was titrated by EIT than when it was titrated by the ARDSnet protocol (27.93 ± 7.65% vs. 33.07 ± 10.57%, *P* = 0.027), as was the center of ventilation (CoV%; 36.0 ± 10.69 vs. 42.21 ± 11.78, *P* = 0.005). There was no significant difference in ventilation distribution in ROI 1%, ROI 2%, or ROI 4% according to the titration method used (Table 4) (Fig. 3).

**Table 4:** Effects of PEEP level on local mechanical distribution in patients with chronic obstructive pulmonary disease according to titration method used

	EIT	ARDS net	*T*	*P*-value
ROI 1%	13.64 ± 5.87	12.0 ± 6.97	2.140	0.052
ROI 2%	49.93 ± 7.03	47.36 ± 10.07	1.706	0.112
ROI 3%	27.93 ± 765	33.07 ± 10.57	2.507	0.027
ROI 4%	8.071 ± 4.63	9.14 ± 4.22	1.041	0.317
CoV%	36.0 ± 10.69	42.21 ± 11.78	3.405	0.005

*CoV*% center of ventilation, *EIT* electrical impedance tomography, *ROI* region of interest

#### Oxygen metabolism

Oxygen delivery was significantly greater when PEEP was titrated by EIT (466.40 ± 71.08 mL/min/m^2^ vs. 411.10 ± 69.71 mL/min/m^2^, *P* = 0.001). However, there was no significant difference in central venous oxygen saturation regardless of the PEEP titration method (*P* > 0.05) (Table 3).

## Discussion

The main findings of this study were that (1) the PEEP level, titrated by both EIT method and ARDSnet protocol, was lower in ARDS patients with COPD as compared to patients without COPD and (2) the PEEP level selected by EIT method was lower in ARDS patients with COPD as compared to that titrated by the ARDSnet protocol.

This is the first study to optimize titration of PEEP by EIT and the ARDSnet protocol in ARDS patients with COPD. These patients have obvious PEEPi and greater lung compliance and EELV than their counterparts without COPD. A questionnaire-based study by Rose et al. [22] showed that the PEEP level required in mechanically ventilated patients with acute exacerbation of COPD was lower than that needed in patients with ARDS but higher than that in patients requiring mechanical ventilation after surgery. A lung-protective ventilation study showed that patients with COPD requiring mechanical ventilation had a higher PEEP level than those without underlying pulmonary disease but a lower PEEP level than patients with ARDS and no comorbidities [23]. The pathophysiological characteristics in the lungs of patients with ARDS and COPD may be different from those typically seen in patients with ARDS alone. The potential negative effects of application of PEEP include increased risks of PEEPi and dynamic pulmonary hyperinflation, which may be more severe in patients with acute exacerbation of COPD requiring mechanical ventilation. Our study showed that the PEEP titrated by EIT was significantly lower than the PEEP selected by ARDSnet table in ARDS patients with COPD. While keeping the PaO_2_/FiO_2_ and PH stable, the ARDS patients with COPD were ventilated with lower PEEP; thus, the lungs received a lower mechanical power. Moreover, the possible benefit of PEEP titrated by EIT in such patients is that it allows the alveoli to maintain in an open state without a significant increase in the proportion of lung tissue that is excessively expanded. Therefore, EIT, which helps to avoid excessive inflation, worsening dyspnea, and hemodynamic disturbances, may be more suitable for titration of PEEP in patients with ARDS and COPD [24].

We found no statistically significant difference in improvement of oxygenation whether PEEP was titrated by EIT or the ARDSnet protocol in patients with ARDS and COPD. The peak airway pressure, plateau airway pressure, VR, mechanical power, Vd/Vt, and EELV were lower, and lung compliance was better when EIT was used, which indicates that the respiratory mechanics indices used by EIT are better than those used in the ARDSnet protocol.

Mechanical power (MP) was found to be independently associated with mortality, ventilator-free days, ICU and hospital length of stay. Furthermore, they showed a consistent increase in the risk of death with MP higher than 17.0 J/min [25]. As an important component of mechanical power [26], PEEP contributes to the mechanical power required to ventilate the lung and sustained PEEP contributed to potentially lethal lung injury and hemodynamic impairment above a threshold level of mechanical power [27]. We found that the PEEP titrated by EIT-based method was lower than it was titrated by the traditional method which resulted in a lower mechanical power delivered to the lung.

Intrapulmonary shunt may develop as a consequence of alveolar atelectasis, and adequate PEEP is necessary to prevent alveolar atelectasis, decrease shunt and improve oxygenation. In this study, it was found that ARDS patients with COPD had significant intrapulmonary shunt, and the shunt fraction was similar to that of COPD patients with respiratory failure requiring mechanical ventilation [28]. Optimal PEEP and prone position are both capable of reducing shunt fraction of ARDS patient [29]. We found no significant increase in shunt fraction when ARDS patients with COPD were ventilated with a lower PEEP selected by EIT method, which further confirmed the lower level of PEEP did not increase the risk of alveolar atelectasis. Furthermore, Sinha et al. [30] retrospectively investigated 1307 patients with ARDS using VR, one of the indicators of ventilation efficiency at the bedside. They found that patients with a higher VR had an increased risk of death, indicating that the VR could be used as an indicator of the mortality risk. In another study by Sinha et al. [31], VR was significantly associated with dead space ventilation and was an independent predictor of mortality. Improvements in respiratory mechanics may help to improve patient outcomes. The findings of our present study show improvement of respiratory mechanics and lower MP when patients ventilated with the PEEP level titrated by EIT than by the ARDSnet protocol which may be beneficial in terms of the prognosis in ARDS patients with COPD.

Another aspect of the application of PEEP in patients with ARDS is its impact on cardiac function. Our study found that the cardiac index was higher and the dose of vasoactive therapy was lower when EIT was used instead of the ARDSnet protocol. High PEEP levels may reduce the cardiac index by increasing intrathoracic pressure, impeding venous return, and possibly increasing pulmonary vascular resistance by compressing alveolar vessels. In an early clinical study of PEEP, Suter et al. [32] assessed the effect of different PEEP levels on the cardiac index. However, no lung-protective ventilation strategy was used in that study. Dantzker et al. [33] investigated the relationship between cardiac output and mechanical ventilation-related intrapulmonary shunt in 20 patients with ARDS and found that high PEEP or tidal volume ventilation resulted in increased shunting and decreased cardiac output. These authors pointed out that hemodynamic changes need to be taken into account when considering improvements in gas exchange in patients with ARDS. A study by Barthélémy et al. in patients with COVID-19-related ARDS found a gradual decrease in cardiac output with increasing PEEP level [6]. In another report, Mercado et al. suggested that lung recruitment and high PEEP ventilation caused a decline in cardiac function, especially in right ventricular function [34]. Other researchers have found that about 25% of patients with ARDS who were mechanically ventilated developed pulmonary hypertension or right ventricular insufficiency even if lung-protective mechanical ventilation strategy was used [35], and right ventricular insufficiency is a risk factor for death in these patients [36, 37]. Elevated PEEP levels cause marked decrease in cardiac output in patients with cardiac disease [16] and COPD is a risk factor for right ventricular dysfunction [17]. In our study, two-thirds of patients with ARDS and COPD had right ventricular insufficiency. PEEP titration by EIT reduced the risk of decreased cardiac function.

In this study, oxygen delivery in ARDS patients with COPD was greater when PEEP was titrated by EIT than by the ARDSnet table. Suter et al. [32] identified oxygen delivery as the variable that provides the best compromise for reconciling oxygenation requirements and hemodynamics. In the critical state of acute hypoxemia that occurs in ARDS, oxygen consumption increases, oxygen delivery decreases, and tissue hypoxia leads to a series of vicious circles. One of the main treatment goals in patients with ARDS is to increase oxygen delivery and improve tissue hypoxia. The use of oxygen-related indicators, including oxygen delivery and consumption, is now used widely as clinical indicators of hypoxia in critically ill patients. Many patients with ARDS have pathological oxygen dependence [38, 39], alteration of which is also a key goal of mechanical ventilation in ARDS. Studies have shown a relationship between maintenance of adequate oxygen delivery and a good prognosis. Maintenance of oxygen delivery at approximately 600 mL/min/m^2^ can reduce complications and shorten the hospital stay after surgery in patients with ARDS [40]. In our present study, oxygen delivery was improved, without increasement of FiO_2_ or transfusion of red blood cells, only by the use of EIT as the PEEP titration method. This may be attributed to the improvement of respiratory mechanics and circulatory function by EIT.

Before 1998, there were few studies on comorbidities in patients with ARDS. Zilberberg et al. [41] prospectively observed the comorbidities of ARDS (including COPD) and identified patient age and the etiology of ARDS to be independent predictors of in-hospital mortality. Azoulay et al. [42] have reported a multicenter prospective observational study that spanned 17 years and included 4953 patients with ARDS, of whom 51.4% had severe comorbidities, including COPD, chronic cardiac insufficiency, and tumors. In that study, the most common comorbidity was COPD (*n* = 948), and ARDS had a mortality rate of 27.2% in patients without comorbidities and 31.1–56% in those with comorbidities. These findings require more attention and inclusion in randomized controlled studies of patients with ARDS and severe comorbidities. In the future, with improved life expectancy, patients with ARDS and chronic complications will become increasingly common in clinical practice. However, reviewing the large-scale clinical trials of mechanical ventilation in ARDS published after 2000, when lung-protective ventilation strategies such as small tidal volume ventilation were first promoted, the inclusion criteria for most clinical trials did not include ARDS patients with COPD [36, 43], which may greatly reduce the ability to generalize the results of clinical research. In recent years, the deepening understanding of the pathophysiology of ARDS has led to further development and refinements of mechanical ventilation in patients with ARDS. Currently, it is believed that the application and management of ventilation in patients with ARDS should be based on individual pulmonary pathophysiological changes to improve the prognosis [1, 44]. The PEEP setting should be individualized based on indicators such as gas exchange, hemodynamics, recruitment potential, end-expiratory transpulmonary pressure, and driving pressure [45]. Individualized lung-protective ventilation based on pathophysiological changes may be an important factor in improving patient outcomes.

At present, various EIT methods-based PEEP titration have been reported in ARDS patients, including overexpansion and collapse (OD/CL) method [10], end-expiratory lung impedance (EELI) method [46], GI index method [14] and regional ventilation delay (RVD) method [47] and so on. Our study proved the feasibility of PEEP titration based on GI index in the ARDS patients with COPD. There are differences in "optimal PEEP" titrated by different PEEP methods [48] and “best” EIT parameter for PEEP titration in mechanical ventilation is still undetermined. Combination of two or more EIT-based parameters for PEEP titration may present a promising tool. Clinical studies have supported OD/ CL method and GI as common reference index in animal study and respective clinical cases study in ARDS [14, 49].

Severe COPD is characterized with expiratory flow limitation and dynamic hyperinflation (DH), resulting in intrinsic positive end-expiratory pressure (PEEPi), increased work of breathing, ventilation heterogeneity, and hemodynamics compromise. Application of optimal PEEP is helpful to reduce airway resistance, PEEPi, and lung hyperinflation. EIT has proven to be useful tool to optimize the PEEP to overcome gas trapping and DH. Regional ventilation delay (RVD) and end‑expiratory lung impedance (EELI) were used to guide the optimization of the PEEP in COPD [50, 51]. Kostakou et al. showed that setting PEEP at 80% iPEEP achieved lowest RVD, highest the expiratory tidal volume and lowest the airway resistance in a patient with severe acute COPD exacerbation [50]. In a prospective exploratory study, Karagiannidis et al. developed and validated an EIT-based method to measure regional expiratory time constants (*τ*) on a breath-by-breath basis and pixelwise level. They found that a widespread inhomogeneous frequency distribution of regional *τ* values ranging from 2 to 5 s in patients with COPD, indicates a huge variation in spatial distribution of *τ*. Moreover, different PEEP levels were shown to have an influence on the distribution pattern of regional *τ* in COPD patients. Thus, *τ* determined by EIT provides a promising tool to individually adjust the level of PEEP in response to the patterns of regional airflow obstruction [5]. Therefore, EIT measure which can provide spatial and temporal distribution of airflow limitation in response to different PEEP settings is helpful to optimize the external PEEP in patients with severe COPD or other obstructive pulmonary disease.

This study has some limitations. First, it was performed at a single center, and the study population was small despite which is comparable to other physiologic studies in the field [52–54]. Therefore, our findings must be considered preliminary. Second, we did not compare the effects of the two PEEP titration methods according to duration of mechanical ventilation. Multicenter prospective randomized trials that include larger sample sizes are needed in the future to explore regional ventilation distribution and regional blood perfusion under different PEEP levels.

## Conclusion

In this study, we found that titrated PEEP levels were lower in ARDS patients with COPD than in ARDS patients without COPD. PEEP titrated by EIT method was lower than that titrated by the ARDSnet protocol in ARDS patients with COPD, and ventilation with PEEP titrated by EIT method shows significant improvements in the ventilation ratio, mechanical power, cardiac index, and oxygen delivery, and had less adverse impact on hemodynamics.

## Supplementary Information

**Additional file 1: Table S1.** Description of clinical characteristics of COPD patients.

## Data Availability

The dataset used to generate this manuscript may be made available from the corresponding author on reasonable request.

## Abbreviations

COPD: Chronic obstructive pulmonary disease
ARDS: Acute respiratory distress syndrome
PEEP: Positive end-expiratory pressure
EIT: Electrical impedance tomography
VILI: Ventilator-related lung injury
GI: Global inhomogeneity
SpO_2_: Peripheral blood oxygen saturation
EELV: End-expiratory lung volume
VR: Ventilation ratio
CoV: Center of ventilation
ROI: Region of interest
