# Supplementary material for: Electrical impedance tomography for titration of positive end-expiratory pressure in acute respiratory distress syndrome patients with chronic obstructive pulmonary disease
Source: Crit Care. 2022 Nov 4;26:339. doi: 10.1186/s13054-022-04201-y (PMC9635124; doi:10.1186/s13054-022-04201-y)
Supplement: Supplementary file 1 — Additional file 1: Table S1. Description of clinical characteristics of COPD patients. [file 13054_2022_4201_MOESM1_ESM.docx]

Supplementary table. Description of clinical characteristics of COPD patients.

| Patient NO | COPD  history | FEV1/FVC | pulmonary emphysema by HRCT | Expiratory flow limitation in mechanical ventilation | PEEPi | Inhalational Drug | home oxygen therapy | home NIV |
| --- | --- | --- | --- | --- | --- | --- | --- | --- |
| 1 | Yes | unavailable | Yes | Yes | Yes | Aminophylline | No | No |
| 2 | Yes | 52% | Yes | Yes | Yes | Beta 2 receptor agonist and corticosteroids | Yes | Yes |
| 3 | Yes | 62% | Yes | Yes | Yes | Beta 2 receptor agonist | Yes | No |
| 4 | Yes | 65% | Yes | Yes | Yes | Beta 2 receptor agonist and corticosteroids | Yes | Yes |
| 5 | Yes | 40% | Yes | Yes | Yes | Beta 2 receptor agonist and corticosteroids | Yes | Yes |
| 6 | Yes | 65% | Yes | Yes | Yes | Tiotropium bromide and corticosteroids (oral) | Yes | Yes |
| 7 | Yes | 68% | Yes | Yes | Yes | Tiotropium bromide and corticosteroids | Yes | No |
| 8 | Yes | unavailable | Yes | Yes | Yes | Tiotropium bromide and corticosteroids | No | No |
| 9 | Yes | unavailable | Yes | Yes | Yes | Tiotropium bromide | No | No |
| 10 | Yes | 66% | Yes | Yes | Yes | Aminophylline | No | No |
| 11 | Yes | 60% | Yes | No | Yes | Tiotropium bromide | No | No |
| 12 | Yes | unavailable | Yes | No | Yes | corticosteroids | No | No |
| 13 | Yes | unavailable | Yes | Yes | Yes | Aminophylline | No | No |
| 14 | Yes | 48% | Yes | Yes | Yes | corticosteroids | Yes | No |

FEV1: Forced expiratory volume in the first second, FVC: Forced vital capacity, COPD: Chronic Obstructive Pulmonary Disease, HRCT: High-resolution computed tomography, PEEPi: Intrinsic positive end expiratory pressure, NIV: noninvasive ventilation
